# Technical Aspects and Considerations of Meso-Rex Bypass Following Liver Transplantation With Left Lateral Segment Grafts: Case Report and Review of the Literature

**DOI:** 10.3389/fped.2022.868582

**Published:** 2022-04-25

**Authors:** Christina Dalzell, Paola A. Vargas, Kyle Soltys, Frank Di Paola, George Mazariegos, Nicolas Goldaracena

**Affiliations:** ^1^School of Medicine, University of Virginia, Charlottesville, VA, United States; ^2^Division of Transplant Surgery, Department of Surgery, University of Virginia Health System, Charlottesville, VA, United States; ^3^Department of Surgery, Hillman Center for Pediatric Transplantation, UPMC Children’s Hospital of Pittsburgh, University of Pittsburgh School of Medicine, Pittsburgh, PA, United States

**Keywords:** extrahepatic portal vein obstruction (EHPVO), living donor liver transplant (LDLT), left lateral segment graft, meso-Rex bypass, pediatric surgery

## Abstract

In pediatric patients with extrahepatic portal vein obstruction and complications of portal hypertension, but with normal liver function, a meso-Rex bypass (MRB) connecting the superior mesenteric vein to the intrahepatic left portal is the favored surgical management. Pediatric patients with a history of a partial liver transplant (LT), especially living donors, are at greater risk for portal vein complications. Hence, an adequate knowledge of this technique and its additional challenges in the post-LT patient setting is crucial. We provide an overview of the available literature on technical aspects for an MRB post-LT. Preoperative considerations are highlighted, along with intraoperative considerations and postoperative management. Special attention is given to the even-more-demanding aspect of performing an MRB post-liver transplantation with a left lateral segment. Surgical alternatives are also discussed. In addition, we report here a unique case in which this surgical technique was performed on a complex pediatric patient with a history of a living-donor LT with a left lateral segment graft over a decade ago.

## Introduction

The meso-Rex bypass (MRB), first described by de Ville de Goyet et al. involves using a vein conduit to direct flow from the superior mesenteric vein (SMV) to the left portal vein within the Rex recess in those patients with extrahepatic portal vein obstruction (EHPVO) but an otherwise normal intrinsic liver function (1, 2). It is a common approach to deal with the portal hypertension complications derived from a portal vein thrombosis (PVT) following liver transplantation in those pediatric patients with otherwise functioning liver grafts. It differs from the other mainstay of treatment, a portosystemic shunt (PSS), because blood flow is still directed from the splanchnic system through the non-cirrhotic liver, which may benefit pediatric patients who are still undergoing growth and development (1). This approach requires a profound knowledge of the extrahepatic and intrahepatic anatomy. In this regard, the Rex recess can be identified by its relationship to specific landmarks—it lies within the umbilical scissure between segments II, III, and IV and the anterior part of the recess ends in the umbilical ligament. Understanding this anatomy provides surgeons with the opportunity to have this technique in their arsenal ready to be used when needed (2).

In the non-transplant patient, the most common cause of EHPVO is umbilical vein catheterization in the neonatal period (3). Other causes include previous abdominal surgeries and infection (2, 3). It is less likely related to coagulation disorders than its counterpart in adult patients, and the cause of thrombus formation can also be idiopathic in some cases (2). The main indications for surgical management of EHPVO include complications of portal hypertension such as splenomegaly, severe thrombocytopenia, and variceal bleeding (1, 4).

The cases of EHPVO after liver transplantation (LT) have been previously described in the literature (5, 6). Vascular complications, including portal vein obstruction (PVO), have been found to be associated with living donor liver transplantation (LDLT), especially when performed for biliary atresia (7–10). Furthermore, the creation of an MRB in pediatric patients who have received a partial graft has also been described in the literature (6, 11–15). However, MRB creation after LDLT with a left lateral segment (LLS) is associated with unique challenge due to the loss of important landmarks (6). In this study, we aim to gather and present the technical aspects of MRB in the post-LT setting. In addition, we describe our experience with MRB in a 15-year-old girl with a history of LDLT with an LLS graft when she was 9 months old presenting with EHPVO and life-threatening complications of portal hypertension over a decade after LT.

### Preoperative Considerations

Planning an MRB creation post-LT requires a multidisciplinary evaluation of the patient. An important facet of preoperative workup is to exclude the presence of intrinsic liver disease and confirm anatomy using both non-invasive and invasive imaging techniques (16). Liver biopsy is often required to rule out intrinsic liver disease as a cause of portal hypertension (2). Hypercoagulable states or disorders must also be excluded with coagulation labs including commonly inherited hypercoagulable disorders (2). Surveillance esophagogastroduodenoscopy (EGD) should be performed to manage varices preoperatively, especially after a recent bleed (2). Preoperative abdominal imaging is necessary to confirm the patency of the mesenteric vein and left portal vein. This can been achieved with either abdominal ultrasound (US) with Doppler or low-dose postcontrast portal venous phase computed tomography of the abdomen (16, 17). In some cases, MRI or MR venography can also be used to better visualize the SMV-portal vein confluence (16, 17). The patency of the internal jugular vein (IJV), if intended to be used as jump graft, should also be confirmed with US preoperatively. In cases where the anatomy remains difficult to visualize due to variants, post-transplant, or collateralization, transjugular wedged hepatic venous portography is also an important technique (16, 17). This technique is invasive and is performed by interventional radiology. After right internal jugular (IJ) access is obtained, a catheter is directed into the left hepatic vein and a balloon is used to “wedge” and prevent outflow. This allows for retrograde portal venography *via* the injection of contrast (17). Pressure measurements can also be obtained during this procedure, including right atrial, caval, wedged hepatic vein and free hepatic vein pressures.

### Intraoperative Considerations

In terms of the operation, the patient is positioned supine with the head and neck slightly facing right in order to have access to the left IJ, which is usually the preferred side for vein grafting. The IJV is the preferred vascular autograft due to excellent long-term patency rates when compared to unrelated or prosthetic grafts (6). The surgery can be performed through a midline or subcostal laparotomy (2). The approach followed will defer slightly if the creation of the MRB is in a patient with no previous liver surgery vs. a post-transplant patient. Likewise, the approach use for a full graft liver will defer from the approach used for a partial liver given that the usual anatomical landmarks are lost in the latter, making this procedure more challenging in these circumstances. The typical steps followed when creating an MRB in a patient with no previous abdominal surgeries include dividing the round ligament and falciform ligament to gain access to the umbilical scissure and identifying the Rex recess (2). Once the umbilical scissure is dissected, the intrahepatic left portal vein is identified and dissected. For this, it is crucial to resect the liver parenchyma bridging the scissure. This will not only allow exposure and access to the intrahepatic left portal vein but will also avoid the compression of the bypass and ensure a good patency of the graft postoperatively. The intrahepatic left portal vein is dissected for a length of approximately 3 cm on its ventral and lateral aspects, and all small branches taking off the Rex recess into segments 2, 3, and 4 of the liver are dissected and encircled with vessel loops in order to be safely occluded when the anastomosis is pursued. Next, a mesocolic window is created to expose the SMV. The left IJV, the preferred autologous vein graft for the MRB, is procured. The anastomoses are created using 7-0 absorbable Prolene sutures (2). The first anastomosis is typically performed to the Rex recess. A small Satinsky clamp is placed in the Rex recess, and the central portion of the intrahepatic left portal vein is anastomosed end to side to the vein autograft in a running fashion. The clamp is released to ensure that the bypass fills with blood from a patent intrahepatic system. Later, the distal end of the jump graft vein is positioned across the mesocolon and anastomosed to the SMV in an end-to-side fashion (2, 18).

The main difference when performing this procedure in a transplanted patient with a left lateral partial graft is that the anatomical references and landmarks that allow us to easily identify and locate the rex Recess are basically lost. Therefore, preoperative imaging and workup play a key role as a route map. Also, once the hilar structures are identified, one should stay above the biliary plate and understand that the Rex will be in close proximity to the cut edge of the liver that, due to hypertrophy, has rotated to the right upper quadrant.

### Postoperative Management

Postsurgical imaging includes Doppler US to confirm bypass patency and measure flow (17). This is typically performed at postoperative days 1 and 3 and then at monthly intervals until 6 months post-bypass creation (17). Later, US are adequate only if clinically demanded due to new signs of portal hypertension (17, 19). CT and MRI can be used to supplement in cases with complex anatomy or if there is a concern for bypass patency (19). Anticoagulation remains a hallmark of treatment after bypass creation (2). In a study by Bhat et al. focused on evaluating the postoperative anticoagulation factors associated with bypass thrombosis, warfarin use was more common in pediatric patients with thrombosed bypass versus open bypass [63% versus 20%; OR 6.5 (95% CI 1.3–315) *p* = 0.022] (20). Thus, heparin is typically used in the immediate post-transplant period until the patient can be discharged on an oral regimen (20). Aspirin and dipyridamole have been reported in the literature, with patients typically discharged on a 3–6-month regimen (2, 6). In our experience, once the patient can tolerate orally, we start them on 81 mg of aspirin daily to maintain for life as the only antithrombotic treatment/prophylaxis.

## Meso-Rex Post-LT

### Meso-Rex Post-LT With Whole Liver Grafts

**FIGURE 1:**
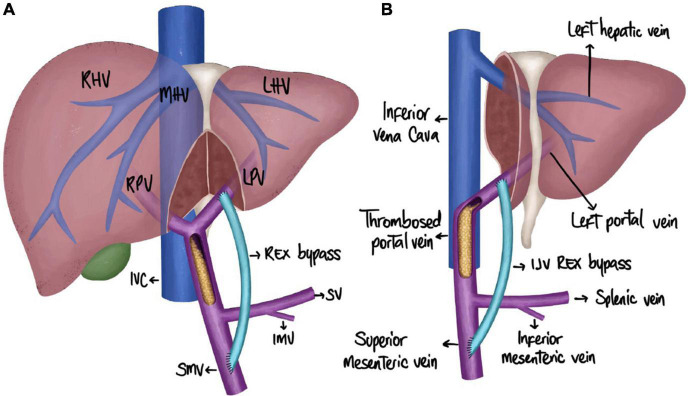
Schematic representation of an MRB creation. **(A)** Meso-Rex post-LT with whole liver grafts. **(B)** Meso-Rex post-LT with partial grafts (LLS). IJV, internal jugular vein; IMV, inferior mesenteric vein; IVC, inferior vena cava; LHV, left hepatic vein; LPV, left portal vein; MHV, middle hepatic vein; RHV, right hepatic vein; RPV, right portal vein; SMV, superior mesenteric vein; SV, splenic vein.

The creation of an MRB following LT comes with unique challenges. MRB creation after a whole deceased donor liver transplantation (DDLT) has been documented in a few case reports and case series (5, 11, 21–23). In an early series by de Ville de Goyet et al. five pediatric patients who underwent LT with a whole graft and developed PVT underwent a successful MRB creation (11). In a case report by Han et al. an adult DDLT recipient underwent MRB creation for portal vein cavernous malformation complicated by hypersplenism and elevated hepatic enzymes (21). A fresh iliac deceased-donor venous allograft was used to connect the left portal vein to the splenic vein. At 6-month follow-up, the bypass remained patent (21). In another case report by Bachman-Braun et al. a pediatric patient with a history of WLT after failed Kasai for biliary atresia developed complications of prehepatic portal hypertension due to PVT (5). She underwent MRB creation with a deceased donor iliac vein. At 6 months after surgery, the patient again developed symptoms of portal hypertension and required surgical revision (5). A large collateral vein was identified and used as an autologous vein conduit to revise the previous bypass (5). In a cohort study by Krebs-Schmitt et al., 14 pediatric patients developed PVT after WLT and underwent MRB creation (22). In this post-LT population, 8 bypass using an autologous jugular vein graft remained patent (22). Two patients who had an autologous jugular vein graft required revision and all 4 patients who had a cryopreserved iliac vein homograft required revision (22). Thus, MRB creation is an effective treatment for EHPVO in both adult and pediatric patients with a history of WLT (Figure 1A).

### Meso-Rex Post-LT With Partial Grafts

Portal vein obstruction is especially common after pediatric LDLT transplantation performed for biliary atresia (10, 24). It is also more common in partial grafts than whole grafts; hence, there are more reports of MRB creation in this patient population (24, 25). A review published in 2012 by de Ville de Goyet et al. includes the instances of MRB creation in reduced or LLS grafts (6). The first case was performed by de Ville de Goyet et al. for a pediatric patient with biliary atresia who received a reduced-size graft (26). From 1992 to 2012, there are reports of 28 pediatric patients undergoing MRB creation with a history of biliary atresia and partial liver transplantation, including reduced-size grafts (segments unspecified) and LLS grafts (6, 11–15, 26). This includes 12 patients who had received an LLS graft from a living-related donor (12, 13, 15). Out of the LDLT patients, 11/12 had long-term survival (12, 13, 15). One patient passed away due to pulmonary sepsis on postoperative day 50 (15). One bypass revision was also required in a patient who received an unrelated donor vascular autograft (12). When WLT transplant recipients were also included for a total of 51 cases of MRB creation after LT, the overall patient survival was 96% with a 100% long-term patency rate with the use of the IJV for the bypass (6).

## Case Presentation

Our patient was a 15-year-old girl with a history of a living-related donor liver transplant with an LLS graft at 9 months old for biliary atresia following a failed Kasai procedure. Her postoperative transplant course was complicated by persistent thrombocytopenia and hypersplenism. She underwent partial splenic artery embolization in 2013 for increasing splenomegaly and thrombocytopenia, consistent with splenic sequestration. In 2014, she had an evidence of PVT with cavernous transformation on imaging and presented with a gastrointestinal (GI) bleeding consistent with esophageal varices and portal hypertensive gastropathy on endoscopy. Since that initial episode in 2014, she underwent repeat surveillance EGD with banding but had no further episodes of bleeding. Her screening endoscopy result in early 2021 showed continued portal hypertensive gastropathy. The computed tomography scan of her abdomen and pelvis 4 months prior to MRB creation showed splenomegaly, measuring 15.6 cm craniocaudal. Preoperative lab results included a platelet count of 81.000. Due to her risk of GI bleeding due to continued portal hypertension, a decision was made to proceed with MRB creation (Figure 1B).

As part of her preoperative evaluation, the patient underwent portal venogram and transjugular liver biopsy, which showed no signs of intrinsic liver disease. Other aspects of preoperative evaluation included echocardiography and an US of bilateral IJVs and subclavian veins.

**FIGURE 2:**
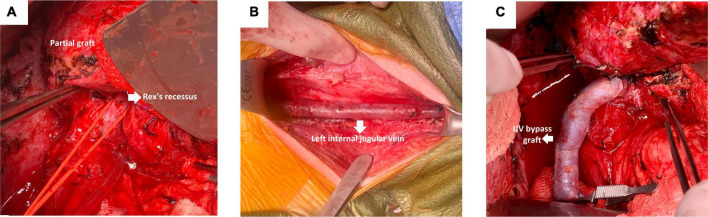
Important steps during an MRB creation. **(A)** Identification of the hilar structures. **(B)** Exposure of the left IJV. **(C)** Anastomosis of the left portal vein and proximal end of the left IJV.

The patient was taken to the operating room for MRB creation (Figure 2). A bilateral subcostal incision was made. The LLS graft was hypertrophied and was mobilized and dissected off the right abdominal wall. The hilar structures were identified (Figure 2A), and a very small bridge of liver parenchyma connecting segment 3 and the small portion of segment 4 was found. This area was carefully dissected to identify the left portal vein (Rex recess). The portal plate and vein were subsequently exposed, and individual branches draining into the portal vein were isolated and protected with vessel loops. Next, the SMV was identified at the root of the mesentery.

The SMV was dissected and exposed distal to the lesser arcuate branches and proximal to the middle colic vein. Next, an incision was made along the anterior border of the left sternocleidomastoid muscle to expose the left IJV. The vein was taken as proximal as possible, down to its takeoff from the subclavian vein (Figure 2B).

**FIGURE 3:**
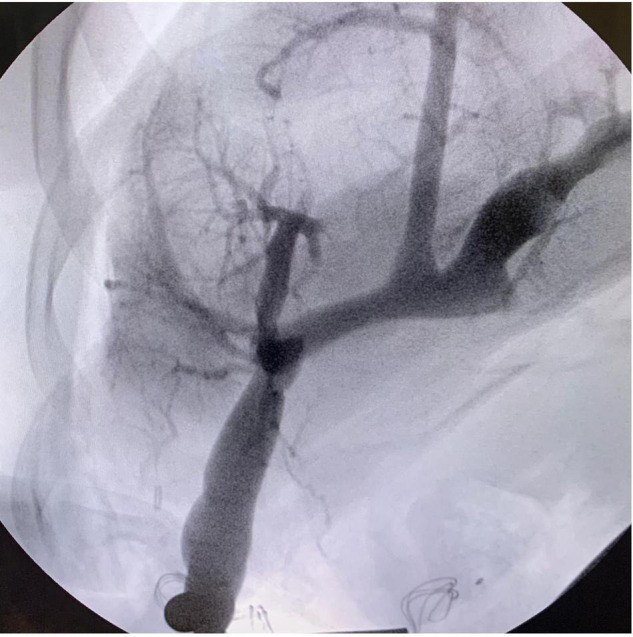
Portal venography via the anastomosed graft with evidence of good flow into the liver.

The following step consisted of the creation of the meso-Rex bypass anastomosis. The portal plate was opened with scissors, and good back bleeding was noted prior to starting the anastomosis. The anastomosis of the left portal vein and proximal end of the left IJV was constructed in a running end-to-side fashion with 7-0 Prolene (Figure 2C). Portal venography was performed and confirmed good flow into the liver from the bypass graft (Figure 3). The graft was tunneled through a hole in the mesentery to the prepared area of the SMV. The SMV was opened, and a running end-to-side anastomosis was completed with 7-0 Prolene. The patient tolerated the procedure well, with no immediate postoperative complication and minimal blood loss.

She remained in the hospital for 1 week and was transitioned from a heparin drip (5 units/kg/h) to aspirin 81 mg daily when tolerating the PO intake. On POD #0, 1, and 3, US showed a patent bypass with appropriate flow. By the second postoperative week, her platelet count had increased to 228.000. On a follow-up US 2 months after MRB creation, her spleen size had decreased to 12.9 cm. At more than 8 months after the procedure, the patient’s postoperative course has been uneventful. She remains on aspirin 81 mg daily.

## Discussion

The creation of a MRB in the post-LT is a challenging procedure. Despite its complexity, with a proper interdisciplinary management and comprehensive patient assessment, excellent postoperative outcomes and survival rates can be achieved. We presented the case of MRB creation in a female patient who underwent LDLT 14 years prior with an LLS graft from a living-related donor. MRB creation was pursued due to the complications of portal hypertension related to EHPVO. Her postoperative course has remained unremarkable. In contrast to previous case studies, this is the first reported case in which this surgical technique was performed on an LLS graft from a living donor over a decade after the original transplant.

Biliary atresia is the most common indication for pediatric LT (9). An increase in the incidence of PVT following LT in patients with biliary atresia has been well established in the literature (7, 9, 27). Portal vein hypoplasia is a classic anatomic feature in patients with biliary atresia, which increases the challenge of anastomotic construction and thus the risk for post-transplant PVT (2, 5, 6). PVT is limited to the extrahepatic portal vein in children and can occur acutely after surgery in 5–10% of cases, or years later, often manifesting with signs of portal hypertension (6). A study by Ou et al. focused on evaluating pre-and post-transplant ultrasonographic findings in patients with biliary atresia who underwent LDLT to predict PVO (7). On multivariate analysis, the only pretransplant independent risk factor associated with post-transplant portal vein occlusion was the small main portal vein size <4 mm (*p* = 0.008). This is consistent with another study by de Ville de Goyet et al. showing that the portal vein size <5 mm is associated with portal vein complications on multivariate analysis after LDLT (11).

There are small case series and case reports on MRB creation after pediatric liver transplantation (6). A few cases describe this procedure in an LLS graft after LDLT. In a series by Gibelli et al. two patients with biliary atresia underwent LDLT and developed PVT on postoperative day 1. They subsequently underwent bypass creation on the same day (15). In a case report by Rivera et al., MRB creation was performed in a 13-month-old baby during the living-related donor transplant operation due to intraoperative PVT (13). In another case report by Caruso et al. a female child with biliary atresia underwent LDLT with surgical ligation of the splenic artery at 8 months. She had an episode of GI bleeding at age 5, with an evidence of complete PVT on imaging (28). The patient subsequently underwent MRB creation. However, the bypass thrombosed 4 months later and she had to undergo splenorenal shunt creation (28).

Meso-Rex bypass creation is especially difficult in patients with a partial graft. As evidenced by the case reports above, it is an uncommon procedure. The anatomic criteria, including a patent SMV and Rex recess with thrombus limited to the extrahepatic portal vein, must be met (6). This was confirmed on preoperative portal venography in our patient. Preoperative liver biopsy is also required to exclude cirrhosis before creating an MRB that will bring back blood to the transplanted liver. Bypass creation in an LLS graft is especially difficult because of the loss of landmarks in identifying the Rex recess and due to the route the bypass must take (6). In our patient, over a decade after a failed Kasai procedure and subsequent transplant, the graft was shifted to the right upper quadrant. IJV autograft is preferred, especially in the post-transplant setting, and has been associated with the best patency and overall outcomes, whereas the use of an unrelated donor vein or prosthetic material has been associated with a higher incidence of bypass thrombosis (6, 22).

Meso-Rex bypass creation differs from the other surgical option, a PSS, because it still allows blood flow through the liver. Studies have compared outcomes after the two techniques, with conflicting results (1, 29). In a retrospective study by Lautz et al., a cessation of variceal bleeding occurred in 96% of patients who underwent meso-Rex bypass versus 100% in PSS (29). However, there was a significant improvement in the platelet count, international normalized ratio, and serum ammonia level in the meso-Rex bypass group (29). In a systematic review by Zielsdorf et al., MRB creation was associated with a higher rate of bypass thrombosis (14.1% versus 5.8%; *p* = 0.021) and reoperation for thrombosis or stenosis (11.8% versus 4.1%; *p* = 0.019) versus PSS (1). Importantly, the neurological benefits of restoring portal blood flow to the liver have been established with meso-Rex bypass creation (30). In this study, neurocognitive testing was performed before and 1 year after surgery (30). Both PSS and MRB groups demonstrated similar fluid cognitive ability at initial evaluation; however, only the MRB group showed significant improvement 1 year after the surgery (30). In another study, patients who underwent MRB showed a significant improvement in their height and weight postoperatively (31). The negative association of EHPVO with growth has a multifactorial etiology, including a decrease in nutrient flow to the liver, malabsorption from portal hypertensive gastropathy, and early satiety from splenomegaly (4). These cognitive and growth benefits have led many surgeons to favor MRB over PSS creation in pediatric patients with non-cirrhotic livers.

Portal vein angioplasty with or without stent placement has also been described as an option for portal vein complications in pediatric LDLT (32). In a retrospective study of 75 pediatric patients who underwent LDLT, there were 6 late-onset PV complications. The initial treatment of portal vein stenosis in 4 patients was PTA with stent placement (*n* = 1) and PTA with balloon dilation (*n* = 3). In the remaining 2 patients, the portal vein was unable to be cannulated due to complete obstruction (*n* = 1) and restenosis with total thrombosis after the previous PTA with stent placement (*n* = 1), so they underwent a successful MRB creation (32). Hence, although PTA may be a first-line option in patients with stenotic portal veins, MRB creation is preferred in late-onset portal vein complications due to complete obstruction.

## Conclusion

In summary, MRB creation in patients following LT is a challenging procedure that requires careful preoperative planning with an adequate, multidisciplinary approach and close follow-up. We demonstrated that patients with a long-term history of LDLT, presenting with advanced complications of portal hypertension, could benefit from MRB with an autologous IJV graft. Despite being a technically challenging procedure, we demonstrated an uncomplicated postoperative course with an adequate patient recovery. A MRB creation for chronic PVT with symptomatic portal hypertension is safe and feasible and remains the favored option for pediatric patients, even years after transplant with partial grafts.

## Author Contributions

NG, PV, and CD participated in conception and design of the manuscript, and writing and drafting of the manuscript. KS, FD, and GM participated in critical revision of the manuscript for important intellectual content. All authors approved the final version of the manuscript.

## Conflict of Interest

The authors declare that the research was conducted in the absence of any commercial or financial relationships that could be construed as a potential conflict of interest.

## Publisher’s Note

All claims expressed in this article are solely those of the authors and do not necessarily represent those of their affiliated organizations, or those of the publisher, the editors and the reviewers. Any product that may be evaluated in this article, or claim that may be made by its manufacturer, is not guaranteed or endorsed by the publisher.
